# Molecular Diagnosis of Sexually Transmitted *Chlamydia trachomatis* in the United States

**DOI:** 10.5402/2011/279149

**Published:** 2011-06-12

**Authors:** April L. Harkins, Erik Munson

**Affiliations:** ^1^Department of Clinical Laboratory Science, Marquette University, Milwaukee, WI 53233, USA; ^2^Wheaton Franciscan Laboratory, 11020 West Plank Court, Suite 100, Wauwatosa, WI 53226, USA; ^3^College of Health Sciences, University of Wisconsin—Milwaukee, Milwaukee, WI 53201, USA

## Abstract

Chlamydia, with its *Chlamydia trachomatis* etiology, is the most common bacterial sexually transmitted infection in the United States and is often transmitted via asymptomatic individuals. This review summarizes traditional and molecular-based diagnostic modalities specific to *C. trachomatis*. Several commercially available, FDA-approved molecular methods to diagnose urogenital *C. trachomatis* infection include nucleic acid hybridization, signal amplification, polymerase chain reaction, strand displacement amplification, and transcription-mediated amplification. Molecular-based methods are rapid and reliable genital specimen screening measures, especially when applied to areas of high disease prevalence. However, clinical and analytical sensitivity for some commercial systems decreases dramatically when testing urine samples. *In vitro* experiments and clinical data suggest that transcription-mediated amplification has greater analytical sensitivity than the other molecular-based methods currently available. This difference may be further exhibited in testing of extragenital specimens from at-risk patient demographics. The development of future molecular testing could address conundrums associated with confirmatory testing, medicolegal testing, and test of cure.

## 1. Ecology and Epidemiology of Urogenital Chlamydia

### 1.1. Prevalence and Transmission

**Figure 1:**
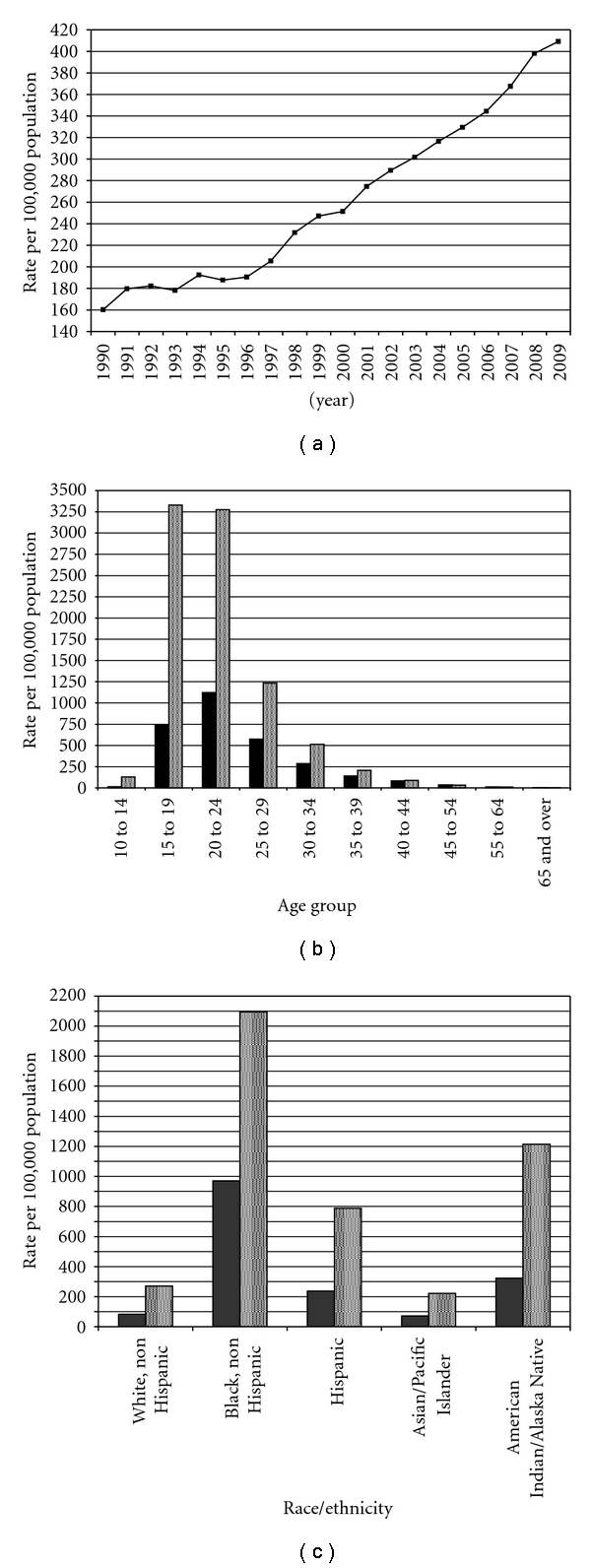
Epidemiology of chlamydia in the United States, summarized in terms of annual incidence rates (a), prevalence within arbitrary age groups (b), and race/ethnicity distribution (c). Solid bars represent male gender and shaded bars represent female gender. Data are adapted from [1].

Since becoming a nationally notifiable disease in the United States in 1995, chlamydia has experienced consistent annual increases (averaging 5.8%) to its 2009 prevalence rate of 409.2 per 100,000 inhabitants (Figure 1(a)), making it the most common bacterial sexually transmitted infection (STI) in this country [1]. Data from the United States Centers for Disease Control and Prevention (CDC) have revealed that chlamydia rates are highest in late adolescents and young adults ([1], Figure 1(b)). African Americans and Native Americans demonstrate higher rates of chlamydia compared to other races or ethnicities (Figure 1(c)).

Sexually active asymptomatic populations have been implicated in widespread transmission of the *Chlamydia trachomatis* etiology. Selective screening of sexually active women has yielded infection rates ranging from 8% to 40% (typical mean of 15%, [2]), while approximately 10% of sexually active asymptomatic males are infected [3, 4]. In contrast to *Neisseria gonorrhoeae* infection in which most patients develop symptoms and seek care promptly, Hook et al. [5] reported that most females and males with *C. trachomatis* infection were asymptomatic or mildly symptomatic upon clinical presentation. Diagnosis was largely on the basis of screening or having a symptomatic contact. Further evidence that chlamydia is a prevalent disease rather than an incident disease comes from extrapolations of STI agent acquisition rates.While past studies have suggested that gonorrhea sexual transmission can be more efficient than chlamydia transmission [6, 7], recent data utilizing *C. trachomatis* molecular diagnostics report less of a disparity between transmission rates [8]. Katz et al. [7] estimated a 0.32–0.39 chlamydia transmission rate when using culture as a detection modality, while Quinn et al. [8] estimated this rate to be approximately 0.68 when utilizing molecular diagnostics. It is important to note that these extrapolations were performed on the basis of historical average frequency of intercourse between pairs, rather than single sexual encounter, which provided the basis for studies of gonorrhea transmission [9, 10].

### 1.2. Urogenital Chlamydia in Males

Ocular trachoma, lymphogranuloma venereum, perinatal infections, and adult oculogenital disease outline four clinical categories of *C. trachomatis* infections described by Stamm et al. [11]. We briefly summarize selected important clinical manifestations of urogenital disease as they pertain to subsequent laboratory diagnosis of the disease etiology. *C. trachomatis* is thought to be responsible for 30–50% of cases of nongonococcal urethritis (NGU) in men. Appropriate laboratory diagnostics in male urethritis are important for at least four reasons: (1) symptom overlap with clinical gonococcal urethritis and NGU may exist (Table 1); (2) prevalence of NGU in the United States exceeds that of gonococcal urethritis [2]; (3) *C. trachomatis* may be detected from a substantial proportion of patients with gonococcal urethritis [12], and concomitantly, (4) dually infected males who are treated solely for gonococcal urethritis are likely to develop post gonococcal urethritis, manifested as persistence or recurrence. Risk factors for chlamydial urethritis have included heterosexual orientation, African American race, and age younger than 20 years [13]. 

### 1.3. Urogenital Chlamydia in Females

Mucopurulent cervicitis caused by *C. trachomatis* is said to be the female counterpart of male NGU, as approximately 70% of women are asymptomatic or experience only mild symptoms such as bleeding, discharge, mild abdominal pain, and dysuria. Being a sex partner of a male with NGU or gonococcal urethritis has been reported to confer an infection risk of greater than 30% [2, 12]. Additional factors such as younger age, African American race, unmarried status, new or multiple sex partners, oral contraceptive use, and residence in the southeast United States promote higher rates of chlamydia in sexually active females [2, 19].

Symptoms relative to mucopurulent cervicitis can also characterize conditions such as cystitis and vaginitis. As such, diagnosis of chlamydia may be masked by diagnosis and treatment of concomitant *N. gonorrhoeae* and *Trichomonas vaginalis* infection. A Milwaukee, Wisconsin laboratory used highly sensitive molecular methods for the detection of these agents [20, 21] to determine the STI profile of 272 female healthcare encounters that proved to be positive for at least one STI. In this populace that ranks second in the United States in both chlamydia and gonorrhea prevalence [1], 17% of patients with detectable *C. trachomatis*-specific nucleic acid had concomitant *N. gonorrhoeae* and/or *T. vaginalis* nucleic acid detection [22]. The development of accurate laboratory diagnostics for *C. trachomatis* bears additional importance in light of data associating chlamydial cervicitis with acquisition of human immunodeficiency virus in women [23, 24].

## 2. Selected Nonmolecular Means of Laboratory Diagnosis

For many years, the accepted gold standard for *C. trachomatis* detection was culture. Culturing techniques in McCoy cell lines are rather complex and time consuming, with the necessity for experienced laboratory technologists for accurate follow-up staining and microscopy. Sensitivity of culture methodology is much less compared to nucleic acid amplification testing (NAAT), allowing for false negative results to potentiate the spread of infection [25–28]. 

In 2002, the CDC recommended routine laboratory screening for individuals at high risk of acquiring STIs [29], particularly with effective treatment regimens for *C. trachomatis* and *N. gonorrhoeae* being both accessible and inexpensive. The detection of *C. trachomatis* by rapid screening or point-of-care methods includes nonamplification methods such as direct fluorescent antibody testing (DFA), optical immunoassay (OIA), and rapid solid-phase enzyme immunoassay (EIA). These methods were developed to provide a level of service to the community, enabling clinicians to begin treating patients on the day of the detection (a “test and treat” strategy) and consequently reducing the risk of inflammatory sequelae and the spread of infection. This represents a significant public health issue as 20% of patients that are diagnosed with *C. trachomatis* do not return to follow-up medical attention within a one-month interval, with 3% of these patients subsequently developing PID within this timeframe [30, 31]. 

The DFA procedure begins with fixing epithelial cells from the conjunctiva, urethra, or cervix to a microscope slide. Monoclonal antibodies specific for *C. trachomatis* major outer membrane protein, conjugated with fluorescein isothiocyanate (FITC), bind to intracellular inclusions if the organism is present. The DFA procedure is considered rapid or point-of-care testing due to its capacity to be performed within 30 minutes, although expertise in reading fluorescence microscopy is required and the method exhibits low sensitivity when compared to NAAT. In one study, Boyadzhyan et al. [32] reported that *C. trachomatis* culture and DFA failed to detect 28% and 0%, respectively, of specimens determined to be positive by NAAT. 

The rapid OIA consists of an optical reading, which has a very subjective color change as its basis. *C. trachomatis* antigens present within specimens will react with specific antibodies impregnated on a silicon wafer. The Biostar OIA has been evaluated in urogenital specimens from women at an STI clinic [25, 26]. Swain et al. [25] evaluated 1,385 women for *C. trachomatis* infection using the DFA, OIA, and culture methods. Sensitivity and specificity for testing methods were 73.6% and 99.9%, respectively, for DFA, 64.2% and 99.1% for OIA, 56.1% and 100% for culture, and 95.3% and 99.8% for PCR. During the study, these modalities were referenced against an expanded gold standard, which included NAAT testing if the culture was negative. The researchers concluded that a universal screening program utilizing rapid testing for laboratory diagnosis of *C. trachomatis* was not recommended. The decision analysis the investigators provided did show that the poorly sensitive rapid testing is potentially useful in clinics where patients do not comply to follow-up treatments.

The true sensitivity of nonmolecular diagnostic testing for *C. trachomatis* is predicated on the quality of the reference standard. Those assays that are compared to culture or analogous rapid or point-of-care testing in terms of sensitivity could demonstrate falsely elevated performance characteristics. Bandea and colleagues [27] showed the sensitivity of the Biostar Chlamydia OIA as 78.6% and specificity as 97.2% with culture as the reference standard. This is in contrast to another study [33], utilizing a reference standard based on concordant results from two NAAT modalities that reported sensitivity and specificity for the Biostar OIA at 59.4% and 98.4%, respectively. Moreover, *C. trachomatis* detection by point-of-care EIA methodology has also shown to have decreased sensitivity compared to NAAT. Van Dommelen et al. [34] demonstrated that sensitivity of three rapid EIAs was extremely low (17.1% to 25%) compared to NAAT. 

Using a test with a low sensitivity may result in patients being falsely reassured by a negative test result, potentiating spread of infection and progression of disease to PID or other infertility sequelae [35]. In assessing the proper rapid test to use for *C. trachomatis* diagnosis, the World Health Organization has recently released the ASSURED criteria for rapid STI assays; affordable, sensitive, specific, user-friendly, rapid and robust, equipment-free and deliverable [35]. The optimal context for utilization of point-of-care testing is reflexive followup with NAAT. Until sensitivity of rapid point-of-care testing improves, one has to be very cautious in using nonamplification methods alone, especially in low-prevalence populations in which assays yield positive predictive value of <90%.

## 3. Molecular Means of Laboratory Diagnosis

### 3.1. Commercial Nucleic Acid Hybridization

Nucleic acid hybridization technologies employ oligonucleotide sequences that are designed to anneal to complementary sequences within target nucleic acid. Because of analytical sensitivity issues inherent to nucleic acid hybridization, this paradigm is generally reserved for clinical conditions with a high organism burden [36]. In a study set of 201 cervical specimens, LeBar et al. [37] determined the sensitivity of PACE 2 (Gen-Probe, Incorporated, San Diego, Calif, USA) for the detection of *C. trachomatis*-specific 16S ribosomal RNA to be 82.8% compared to a *C. trachomatis* cell culture reference. 78.0% assay sensitivity was reported in an additional study of 217 cervical specimens [38]. Specificity of *C. trachomatis*-specific PACE 2 was documented at 98.8–99.4% [37, 38]. In a study of male urethral specimens, Kluytmans et al. [39] developed two off-label modifications to the *C. trachomatis* PACE 2 assay that yielded assay sensitivity of 89.5%. Within 398 endocervical specimens, Limberger et al. [40] reported that 19 of 20 *C. trachomatis* cell culture-positive specimens were also PACE 2-positive. This high frequency of concordance was not noted with specimens that were mailed to the laboratory for analogous nucleic acid hybridization for *N. gonorrhoeae* and may therefore reflect the susceptibility of the latter STI agent to conditions of specimen transport. This may be particularly true in light of past data reporting a 99.4% *N. gonorrhoeae*-specific PACE 2 sensitivity in a sampling of 436 cervical or urethral swabs [41]. Taken together, these data begin to portend that decreased sensitivity of nucleic acid hybridization testing for *C. trachomatis* may be linked with a suboptimal cell culture reference method, rather being limited by mitigating factors such as specimen transport.

### 3.2. Commercial Signal Amplification

Neither target nor oligonucleotide probe nucleic acid concentrations change in the signal amplification paradigm. Instead, the concentration of reporter molecules is increased at the site of target/probe hybridization [36]. Commercially available signal amplification methods detecting *C. trachomatis* alone, or in concert with *N. gonorrhoeae*, utilize hybrid capture technology (Hybrid Capture II (HC2) product line; Digene Corporation (QIAGEN), Gaithersburg, Md, USA). Targets for *C. trachomatis* the detection include specific chromosomal and cryptic plasmid sequences. Schachter et al. [42] evaluated the HC2 CT-ID test using a combined *C. trachomatis* cell culture and direct fluorescent antibody reference method and demonstrated 97.7% sensitivity for detection of *C. trachomatis* from endocervical specimens. Within the 129 true-positive specimens, only 114 (88.4%) yielded a positive *C. trachomatis* culture result. The utilization of the antecedent HC2 CT/GC test to screen for the presence of either *N. gonorrhoeae* or *C. trachomatis* exhibited 95% sensitivity. A two-center study [43] reported 96.6% sensitivity of the HC2 CT-ID test on endocervical specimens from high-risk female populations in relation to a culture reference. However, upon the adjudication of discrepancies with PCR, sensitivity of the HC2 CT-ID test was 97.2% compared to a culture sensitivity of 80.6%. Greater than 98% specificity was noted in both studies [42, 43]. Modarress et al. [44] evaluated the HC2 CT/GC test using genital swab specimens collected for PACE 2 testing and demonstrated approximately 87% and 100% sensitivity of PACE 2 and HC2 CT/GC, respectively, for the detection of either *C. trachomatis* or *N. gonorrhoeae*, although only the difference in *C. trachomatis* detection rate was significant between the two modalities (*P* < 0.016).

### 3.3. Commercial Polymerase Chain Reaction (PCR)

The long-standing PCR [45, 46] DNA target amplification method is the basis of the multiplex AMPLICOR CT/NG product line (including the COBAS fully automated platform), heretofore, referred to as AMP (Roche Molecular Systems, Incorporated, Branchburg, NJ). *C. trachomatis* target for this assay is a 207-nucleotide sequence within a cryptic plasmid that is highly conserved within all serotypes of the organism. Livengood III and Wrenn [28] demonstrated a disparity in the rate of *C. trachomatis* detection from endocervical specimens by AMP (93.3%) versus *C. trachomatis* culture (65.0%)—far greater than that disparity (3.7%) observed for *N. gonorrhoeae*. A multicenter evaluation of AMP yielded 89.2–89.7% sensitivity in the detection of *C. trachomatis* target from female urine and endocervical specimens, respectively, with 88.6–90.3% sensitivity derived from male urethral and urine specimens when using an infected patient standard [47]. Data from a European study [48] demonstrated 92.0–98.0% AMP sensitivity from male specimen sources and female endocervical specimens, yet reported 82.5% sensitivity of *C. trachomatis* detection from female urine specimens. The aforementioned studies reported ≥98.4% specificity from all specimen sources [47, 48]. From a peripheral and foreshadowing sense, noteworthy from the van Doornum et al. data [48] and an additional study [49] was the apparent deficit of AMP to accurately detect *N. gonorrhoeae* DNA from female urine specimens (sensitivity values ranged from 64.8–66.7%). A summary of manufacturer-published performance characteristics of commercial NAAT on urine and endocervical/urethral specimens described in this review is presented in Table 2 [50–52].

### 3.4. Commercial Strand Displacement Amplification (SDA)

A multiplex isothermal DNA target amplification method [53] constitutes a leading diagnostic assay for the detection of *C. trachomatis* in the United States (BD ProbeTec ET *C. trachomatis* and *N. gonorrhoeae* amplified DNA assay (heretofore referred to as ProbeTec); Becton, Dickinson and Company, Sparks, Md, USA). In the context of *C. trachomatis*, SDA targets the chlamydial cryptic plasmid—up to ten copies of which can be found in each cell. A seven-center evaluation [54] utilized *C. trachomatis*-specific cell culture, DFA, and a since-defunct commercial ligase chain reaction to determine infected patient status and related 92.5% ProbeTec sensitivity in the detection of *C. trachomatis* from male urethral swabs. Sensitivity of the assay on male urine (93.1%) exceeded that of the commercial PCR assay described previously [47]. In spite of reasonable sensitivity for the detection of *C. trachomatis* derived from endocervical swabs (92.8%), assay of female urine yielded only 80.5% sensitivity. However, combined percentage specificity of the assay was high among both genders (97.3%, [54]) yet it is noteworthy that the specificity of urine specimens among 124 symptomatic males with positive infection status was 92.6%. The analogous value for urethral specimens was 95.9%. 

### 3.5. Commercial Transcription-Mediated Amplification (TMA)

A third commonly utilized nucleic acid target amplification method in the United States for the detection of *C. trachomatis* has its basis in isothermal TMA [55]. A 10^9^-fold rate of RNA amplification is reported to occur in two hours via TMA [56] in contrast to a 10^6^-fold DNA amplification rate in three to four hours [46]. The multiplex Gen-Probe APTIMA Combo 2 assay (heretofore referred to as AC2) targets *C. trachomatis*-specific 23S ribosomal (r)RNA which is present in high copy number. A seven-site evaluation of 1391 females [21] demonstrated 94.2% *C. trachomatis* assay sensitivity from endocervical specimens. Sensitivity of *C. trachomatis* detection from female urine (94.7%) was markedly higher than those values derived from AMP or ProbeTec. In this study, specificity of AC2 ranged from 97.6% for endocervical specimens to 98.9% for urine specimens. In response to commercial systems, especially AMP, demonstrating nonspecific amplification in the context of *N. gonorrhoeae* NAAT [57–59], direct challenges of AC2 with nonpathogenic *Neisseria* spp. and chlamydiae other than *C. trachomatis* failed to result in amplification [60]. Furthermore, Lowe et al. [61] noted a 10% greater sensitivity of AC2 than AMP for detecting *C. trachomatis* in urine specimens.

## 4. Comparison of Performance Characteristics of Commercial NAAT

### 4.1. Analytical Sensitivity

The increased clinical sensitivity exhibited by AC2 may reflect a phenomenon specific to TMA. A 34.6% serum detection rate of hepatitis C virus (HCV)-specific nucleic acid via TMA was demonstrated in disease relapse patients who had apparent virus clearance according to conventional qualitative and quantitative PCR assays [62]. Sarrazin et al. [63] reported a 51.1% residual serum HCV detection rate by TMA versus conventional qualitative PCR assays, including a 36.4% rate compared to an assay with a lower detection limit of 100 nucleic acid copies/mL. Chernesky et al. [64] prepared mock swab specimens containing propagated *C. trachomatis* elementary bodies and showed that the analytical sensitivity of AC2 was 1000-fold greater than that of ProbeTec and 10-fold greater than that of AMP. AC2 exhibited 100-fold greater sensitivity than the two comparators with analogous mock urine specimens. Ikeda-Dantsuji et al. [65] dispensed standardized amounts of *C. trachomatis* elementary bodies into mock specimens and showed within a subsequent dilution series that AC2 analytical sensitivity was 1000-fold greater than that of AMP. Wood et al. [66] demonstrated a lower limit of detection of *N. gonorrhoeae* via AC2 (10^2^ colony forming units/mL) than that rendered by ProbeTec or AMP (≥10^3^ colony forming units/mL).

### 4.2. Role of Endogenous Specimen Inhibitors

A second contributory factor to the purported increased analytical sensitivity of AC2 is decreased susceptibility of the assay to endogenous inhibitors of nucleic acid amplification. Substances suggested to inhibit *C. trachomatis* NAAT have included hemoglobin, low-pH cervical mucosa, *β*-chorionic gonadotropin, urine crystals, and urine nitrites [67–69]. Analysis of the first-generation Gen-Probe Chlamydia TMA assay using 388 urine specimens revealed an 11.9% rate of amplification inhibition [67]. This figure exceeded that of commercial PCR by nearly 5%. Introduction of the organism-specific nucleic acid target capture protocol with concomitant washing and aspiration (under the auspices of second-generation AC2) has negated this inhibitory effect. Ikeda-Dantsuji et al. [65] subjected mock AC2 and AMP specimens containing *C. trachomatis* near the AMP lower limit of detection to increasing concentrations of phosphate and iron and demonstrated that both chemicals only promoted an inhibitory effect on AMP performance. Gaydos et al. [70] reported 75 true-positive *C. trachomatis* screens from 506 total female and male urine specimens via AC2 plus an additional four specimens that also tested positive by a TMA-based assay targeting an alternative sequence. These data compared favorably to the 72 true-positive results from the same study set identified by ProbeTec.

**Figure 2:**
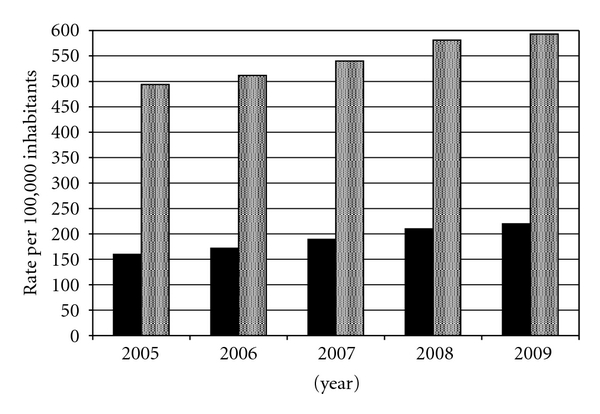
Chlamydia rates per 100,000 inhabitants by gender in the United States from 2005–2009. Filled bars represent male gender and shaded bars represent female gender. Data are adapted from [1].

In an *ex vivo* study [64], rates of *C. trachomatis* nucleic acid amplification inhibition for AC2 (1.3–1.7%) were fairly equivalent to that of ProbeTec (2.0%) for female genital swabs but were far less than those derived from AMP (10.4–12.8%). Rates of amplification inhibition from urine specimens were exceedingly high for ProbeTec and AMP (27.2% and 12.1%, resp.), when compared to AC2 (0.3%). Modifications to the ProbeTec urine collection and transport system have addressed issues related to amplification inhibition [71]. A recent Canadian study [72] reported 98.0% ProbeTec sensitivity in the detection of *C. trachomatis* from 500 urine specimens compared to AC2 sensitivity of 99.0%. Analogous sensitivity indices for the detection of *N. gonorrhoeae* were 95.8% for ProbeTec and 100% for AC2. Improved performance of NAAT on urine specimens, and subsequent overall clinical acceptance of this specimen source, has been cited by the CDC [1] as a factor responsible for a larger increase in *C. trachomatis* detection in males in the United States from 2005–2009 (37.6%) than that increase observed in females (20.3%; Figure 2).

### 4.3. Extragenital Specimen Sources

*C. trachomatis* has a tropism for columnar epithelial cells [73]. This cell type constitutes the vagina of prepubescent girls and is replaced with stratified squamous epithelium upon increased concentrations of estrogen at puberty [74]. In spite of this histological difference, *C. trachomatis* has efficiently been recovered from adult vaginal specimens, further promulgating the high analytical sensitivity of NAAT. Schachter et al. [75] utilized a variety of commercial NAAT modalities to demonstrate that vaginal swabs had nearly equivalent sensitivity to that of endocervical swabs for the detection of *C. trachomatis*, with approximately 12% more sensitivity than first-catch urine. Moreover, patient-collected vaginal swabs had equal sensitivity as clinician-collected vaginal swabs. A multicenter investigation of AC2 performance for *C. trachomatis* on vaginal swabs [76] revealed 96.6% and 96.7% sensitivity on patient- and clinician-collected specimens, respectively, extending previous findings [75]. Positive *C. trachomatis* vaginal screening results were in 91% and 95% concordance with those from endocervical and first-void urine collection, respectively [76]. Sensitivity of *C. trachomatis* AMP from a vaginal swab was 18–22% greater than that of a *C. trachomatis* EIA [77]. *C. trachomatis* detection via ProbeTec and AMP revealed equivalent sensitivity for both vaginal and endocervical specimens [78]. In a study of *C. trachomatis* detection via AC2, 98.6% of infected women were detected via vaginal swab testing, compared to 89.9% and 81.2% from endocervical swabs and first-void urine, respectively [64]. These AC2 specimen-specific percentages of detection were statistically higher than analogous percentages generated by ProbeTec and AMP (*P* = 0.001). In a limited data set (*n* = 25 determinations), our laboratory has demonstrated that the transfer of 200-*μ*L aliquots of vaginal saline suspensions (originally designated for microscopic examination of vulvovaginitis etiologies) into AC2 specimen transport tubes (lysis medium) results in the detection of *C. trachomatis* upon performance of AC2 (E. Munson, unpublished observations). To date, AC2 is the only commercially available modality that has received an FDA indication for vaginal swab collections.

In a description of a potential role for NAAT in laboratory diagnosis of rectal chlamydia, Schachter et al. [79] reported an ≥36.7% increase of sensitivity between NAAT modalities and *C. trachomatis* culture (26.5% sensitivity). In the same males who have sex with males (MSM) demographic, sensitivity of *C. trachomatis* culture from pharyngeal sites was 44.4%. This contrasted with NAAT modalities that reported sensitivity of ≥66.7%. Ota et al. [80] reported sensitivity of *C. trachomatis* culture and two NAAT modalities from rectal specimens as being 21.1% and 94.7%, respectively, from an MSM demographic. The same group reported a significant proportion of pharyngeal detection of *C. trachomatis* via NAAT in the face of a 0% culture-positive rate. Using a rotating infected patient status, Bachmann et al. [81] determined *C. trachomatis* culture sensitivity to be 36.1–45.7% from rectal swabs in a combined MSM and at-risk female demographic.

Early data suggested PCR utility in the detection of *C. trachomatis* from ocular specimens [82, 83], with one report documenting a 26% increase in overall *C. trachomatis* detection over that derived from DFA [83]. Commercial PCR additionally proved to have sufficient diagnostic capacity for ocular chlamydia. Kowalski et al. [84] reported 88.1% sensitivity and 100% specificity of AMP on adult conjunctival specimens. Hammerschlag et al. [85] documented 92.3% sensitivity of AMP derived from infant specimens. Studies have also spoken to the utility of nasopharyngeal and nasal discharge specimens in both diagnosis and predictive value of antimicrobial therapy in the context of chlamydial conjunctivitis [85, 86]. Children with a positive *C. trachomatis* AMP result on a nasal discharge at the commencement of macrolide therapy had an odds ratio of 5.15 to yield a positive AMP result from an ocular specimen two months after therapy when compared to children with a negative AMP result from nasal discharge at baseline [86].

Comparisons of commercial NAAT modalities for the detection of *C. trachomatis* from non-FDA-indicated extragenital sources have ensued. Schachter et al. [76] reported that 32.9% of *C. trachomatis*-positive vaginal screening results obtained by AC2 could not be replicated via ProbeTec analysis of a corresponding first-void urine specimen. 64.7% sensitivity of AMP for the detection of *C. trachomatis* has been demonstrated from rectal specimens when compared to AC2 [80]. The same study reported 33.3% AMP sensitivity for the detection of pharyngeal *C. trachomatis* using a reference infected patient status in what turned out to be a very low-incidence specimen source. Furthermore, AC2 was 30% and 33% more sensitive than ProbeTec in detection of *C. trachomatis* nucleic acid from rectal and pharyngeal sites, respectively. For diagnosis of rectal chlamydia using a rotating infected patient status, Bachmann et al. [81] calculated sensitivity ranges of AMP, ProbeTec, and AC2 at 80.7–95.5%, 92.2–100%, and 100%. Ota et al. [80] reported that AC2 outperformed ProbeTec by 15–20% in an MSM demographic in terms of sensitivity from rectal and pharyngeal specimens, respectively. In further support of this paradigm, in studies of *N. gonorrhoeae* detection from pharyngeal and rectal sites, Bachmann and colleagues [59, 81] determined the performance of AMP to be inferior to that of ProbeTec or AC2. In a study of an MSM demographic [87], of 86 pharyngeal and 99 rectal specimens that generated a positive AC2 result, only 32.6% and 34.3% were positive by *N. gonorrhoeae* culture, respectively. Of the 102 glans specimens positive for *N. gonorrhoeae* by AC2, 96–100% of these results were confirmed by secondary NAAT. In contrast, a higher percentage of *N. gonorrhoeae* AMP-positive rectal swabs were positive by *N. gonorrhoeae* culture when compared to analogous AC2 data. Collectively, these findings challenge the overall analytical sensitivity of AMP for the detection of STI etiologies from pharyngeal and rectal sources.

Limited comparative data exist on molecular detection of *C. trachomatis* from ocular specimens. In an Italian study reported by Fontana et al. [88], overall sensitivity of ProbeTec for the detection of *C. trachomatis* was 76.5% when compared to a laboratory-developed PCR assay targeting 16S rDNA which detected all 34 positive specimens. Two of three *C. trachomatis*-positive ocular specimens were detected by ProbeTec (all three were detected by the assay targeting 16S rDNA). It was noted that a second laboratory-developed PCR assay targeting *C. trachomatis* plasmid DNA detected 28 of 34 overall positive specimens (2 of 3 positive ocular specimens). A *C. trachomatis* plasmid DNA deletion rate of 17.6% was noted in this study. In a study conducted in Ethiopia, Yang et al. [89] demonstrated that a TMA-based assay specific solely for *C. trachomatis* 16S rRNA (ACT; Gen-Probe) had a detection rate of 59% which was in contrast to an AMP-derived 28% detection rate. Increased detection of ocular infection by TMA was independent of active clinical disease (*P* ≤ 0.004). These findings extended those of a previous study [90]. Seven of 15 TMA-positive/AMP-negative specimens had detectable rRNA subsequent to a 1 : 10 dilution of the original ocular specimen. Taken together, these data are relevant because rRNA detection can mitigate the possibility of *C. trachomatis* plasmid deletion [88], low *C. trachomatis* burden has been demonstrated in the context of trachoma management [91, 92], and rRNA concentration far exceeds that of genomic DNA and plasmid DNA in *C. trachomatis* [93].

### 4.4. Trends

**Figure 3:**
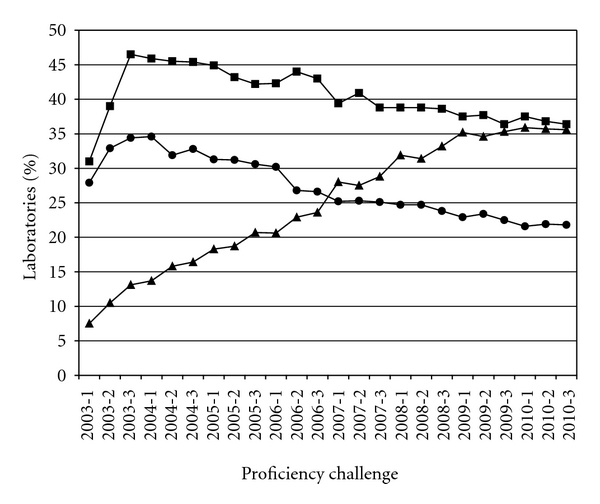
Utilization of AMPLICOR (circles), BD ProbeTec ET (squares), and APTIMA Combo 2 (triangles) platforms for *Chlamydia trachomatis* screening in United States laboratories, as captured by College of American Pathologists proficiency testing participant summary data collections, 2003–2010. Data are adapted from [95].

Several of the aforementioned findings, plus considerations related to the detection of other sexually-transmitted agents [20, 94], may account for increased utilization of commercial TMA for the detection of *C. trachomatis*. Surveys of NAAT modalities employed by clinical laboratories in the United States, conducted by the College of American Pathologists laboratory accreditation program [95], have demonstrated an approximate 30% increase in the utilization of AC2 for *C. trachomatis* screening from 2003–2010 (Figure 3). Overall participant enrollment in these surveys has ranged from 525 laboratories in early 2003 to an average of 925 in 2010. Furthermore, a 2004 survey of United States public health laboratories reported that 87% of respondents performed NAAT for the detection of *C. trachomatis*, while less than 40% offered nucleic acid hybridization. Of the laboratories that offered NAAT, 50% utilized ProbeTec, while 48% performed AC2 [96].

Abbott Laboratories (Des Plaines, Ill, USA) has recently introduced a testing platform (*m*2000) to accommodate both automated specimen processing and real-time multiplex PCR for the detection of regions of the *C. trachomatis* cryptic plasmid and *N. gonorrhoeae* opacity (Opa) gene. Limit of the detection was reported at 20 copies of DNA for each analyte [97]. When assessed against AMP and ProbeTec using residual genital swab material, the *m*2000 demonstrated 96.3–99.1% concordance of positive *C. trachomatis* result, with 98.2–100% concordance of negative result. Urine testing demonstrated high concordance with AC2 and ProbeTec for *C. trachomatis*-negative results (≥98.9%), with 93.7% and 96.8% concordance rates, respectively, for *C. trachomatis*-positive results [97]. Levett et al. [72] compared automated versions of ProbeTec (Viper system) and AC2 (TIGRIS DTS system) to the *m*2000 for *C. trachomatis* urine testing and reported that sensitivity ranged from 96.9% (*m*2000) to 99.0% (AC2).

A strain of *C. trachomatis* with a 377-base pair cryptic plasmid deletion [98] is implicated in the purported decreased rates of positive *C. trachomatis* NAAT results reported in clinical laboratories in Sweden beginning around 2004 [99]. Interestingly, Herrmann et al. reported a proportional rate for this *C. trachomatis* variant ranging from 20% to 64% in regions that utilized either *m*2000 or a commercial real-time PCR system distributed by Roche Molecular Systems. In contrast, in locales that utilized ProbeTec, the proportional rate of the *C. trachomatis* variant ranged from 7% to 19% [99]. Despite these Swedish prevalence data, the variant has been identified from clinical specimens in only two neighboring countries [100].

Amidst concern that the *m*2000 demonstrated poor utility in the detection of European plasmid mutant *C. trachomatis* strains [99, 101], modifications were made to the Abbott Laboratories primer sets. This reformulated product (Abbott RealTime CT) was assessed, along with AC2 and version 2 of the COBAS TaqMan CT test (Roche Molecular Systems), against a panel of 148 *C. trachomatis*-positive urine specimens [102]. Nearly 25% of these specimens contained the variant *C. trachomatis* strain. Assay specificity was nearly 100% for all three systems. Sensitivity of the COBAS TaqMan CT test (83.0%) was outpaced by analogous indices for the Abbott Laboratories reformulation (95.3%) and AC2 (99.3%). A separate study [103] reported that the reformulated Abbott Laboratories assay yielded slightly higher sensitivity than ProbeTec.

## 5. Addressing the Issue of Specificity: Confirmatory Testing

### 5.1. Principle and Methods

Efforts to enhance *C. trachomatis* NAAT sensitivity theoretically come at the expense of assay specificity. Overall scenarios that could generate such false-positive results include the nucleic acid target of interest being present within other organisms endogenous to a specimen, the detection system generating signal in the absence of target; iatrogenic contamination, and, clerical errors [29]. In light of this, past literature from the CDC stated that NAAT assays for *C. trachomatis *are indeed screening assays and that an initial positive result should be considered strictly as presumptive evidence of infection. As such, the CDC deemed necessary the verification of a positive screen in cases that could have adverse medical or psychosocial impact [29]. Furthermore, consideration should be given for the verification of positive NAAT screens for analyses performed in low-prevalence STI populations that would render positive predictive values on the order of 90% or less.

CDC-advocated approaches to additional molecular testing have been four-fold: (1) testing a second primary clinical specimen with an assay that utilizes a different target and a different format, (2) testing the original primary clinical specimen with an assay that utilizes a different target and a different format, (3) repeating the original test on the original primary clinical specimen with a competitive probe, and, (4) repeating the original test on the original specimen. Laboratories that choose commercial nucleic acid hybridization as the method of choice for the detection of *C. trachomatis *can utilize a direct and competitive probe-based nucleic acid hybridization technology [39, 40]. However, it is not advisable to utilize less-sensitive signal amplification or nucleic acid hybridization technologies to confirm a positive screen derived by NAAT [29]. The method advocated first and foremost may not be a reality in certain healthcare environments or in the public health sector due, in part, to the difficulty in successfully contacting a patient to return for specimen recollection.

### 5.2. Repeat Testing

Recent literature suggests that the paradigm of repeat testing may introduce difficulties to the final interpretation of NAAT results. Culler et al. [104] reported that 5.3% of initially positive *C. trachomatis* screens yielded by ProbeTec failed to duplicate results upon repeat testing. Castriciano et al. [105] demonstrated that 93.1% of initially positive *C. trachomatis* screens derived by AMP remained positive upon repeat testing of original lysates. This value dropped to 85.3% upon testing a second nucleic acid extraction. Schachter et al. [106] reported that only 83.8% of positive *C. trachomatis* screens derived by ProbeTec retained positive status upon repeat testing. This value was elevated to 92.5% upon a second repeat ProbeTec assay. In contrast, 96.7% and 97.7% of AMP and AC2 screens, respectively, generated a positive result upon repeat testing. This phenomenon has also been noted in molecular detection of *N. gonorrhoeae*. In the study of Culler et al. [104], 10.7% of positive *N. gonorrhoeae* screens obtained via ProbeTec failed to retain positive status upon repeat testing. Using multiple specimen sources, Moncada et al. [87] demonstrated that 89.3% of positive *N. gonorrhoeae* screens derived by ProbeTec retained positive status by repeat testing. This value increased to 92.4% and 93.1% upon a second and third repeat test, respectively. In contrast, repeat testing of positive screens initially derived by AMP and AC2 retained positive status 95.7% and 96.4% of the time, respectively.

### 5.3. Low-Positive Screens

A further delineation of positive NAAT screens reveals an additional conundrum in terms of a role for confirmatory testing in final result interpretation. 80.8% of low-positive *C. trachomatis *screens derived from ProbeTec (defined as signal detection method other than acceleration (MOTA) scores from 2000 to 9999) remained positive upon repeat testing, while only 33.3% of *N. gonorrhoeae* screens retained the positive status [104]. This paradigm may be of greater consideration when studying a highly sensitive assay such as AC2. Two reports [107, 108] documented positive status retention rates of 42–63% for low-positive *C. trachomatis* screens (defined as relative light unit values between 100,000 and 1,000,000). In contrast, Dunham et al. [107] determined that only 31.6% of low-positive *N. gonorrhoeae* screens retested positive. In a high-prevalence population for both *C. trachomatis* and *N. gonorrhoeae*, 71.3% and 58.5% of low-positive *C. trachomatis* and *N. gonorrhoeae* screens, respectively, yielded positive results upon repeat testing [109]. No significant difference existed between the percentages of low-positive *C. trachomatis* and *N. gonorrhoeae* screens that remained positive by repeat testing (*P* = 0.10). Despite the fact that repeat testing potentiates result interpretation challenges, this low-positive phenomenon, even under the auspices of AC2, presents itself in just 2% of *C. trachomatis* screens and less than 0.1% of *N. gonorrhoeae* screens performed [107, 109].

### 5.4. Alternative Target Testing

A fourth CDC-advocated practice, the utilization of an alternative NAAT system or platform, has met with variable success. Schachter et al. [110] reported that while AC2 confirmed 96.9% of positive ProbeTec screens (95.4% in urine specimens, 98.6% in genital swab specimens), ProbeTec was able to confirm only 82.0% of positive *C. trachomatis* screens derived from AC2 (85.3% in urine specimens, 78.9% in genital swab specimens). Chernesky et al. [64] reported 69.6% and 80.3% rates of confirmation of positive AC2 urine *C. trachomatis* screens by AMP and ProbeTec, respectively. Analogous rates for confirmation of positive AC2 endocervical *C. trachomatis* screens were 62.9% and 70.9%. In contrast, 98–100% of positive urine or genital swab *C. trachomatis* screens yielded by ProbeTec or AMP were confirmed by AC2. In a small subset of specimens that tested equivocal for *C. trachomatis* via AMP or yielded a discrepant result in the context of a combined reference standard, Peterson et al. [111] reported that only 23.1% of specimens yielded a concordant result when subjected to separate PCR assays targeting different sequences. A *C. trachomatis* concordance rate of 82.1% was demonstrated from initial nucleic acid extracts when commercial ligase chain reaction-positive urine screens were tested by AMP [105]. These data further substantiate differences in analytical sensitivity of these NAAT modalities. Similar findings were derived from *N. gonorrhoeae* confirmatory testing. Moncada et al. [87] demonstrated that percentages of positive *N. gonorrhoeae* screens derived by ProbeTec that were confirmed by AC2 and AMP analysis were 85.0% and 78.4%, respectively, and that 84.6% of positive AC2 *N. gonorrhoeae* screens were confirmed by ProbeTec. Yet when similar analysis was restricted to male urine specimens, nearly all positive *N. gonorrhoeae* screens, independent of modality, were confirmed by secondary NAAT. 

APTIMA CT and GC assays have allowed for detailed analysis of the CDC-advocated practice of confirmatory testing using an alternative nucleic acid target. Sensitivity and specificity values greater than 96% were demonstrated for these assays in a multicenter study of MSM using both urethral swabs and urine specimens [112]. Boyadzhyan et al. [32] reported complete concordance of AC2 *C. trachomatis* and APTIMA CT assay results on 253 urine specimens and 422 genital swab specimens collected from either gender. Schachter and colleagues [106, 110] demonstrated that the APTIMA CT assay confirmed 98-99% of positive AC2 screens, with just slight differences noted between concordance values from urine and genital swab specimens [106]. Comparable data have been reported with respect to *N. gonorrhoeae* confirmatory testing. Golden et al. [113] reported that 258 of 265 positive *N. gonorrhoeae* screens of female urine or endocervical specimens derived by AC2 also yielded a positive APTIMA GC assay result. Moncada et al. [87] demonstrated that 95.7% of combined gender specimens initially screening positive by AC2 yielded a positive APTIMA GC result.

### 5.5. Comparisons of Repeat Testing to Alternative Target Confirmation

Data from a five-state United States moderate-prevalence chlamydia population (cumulative *C. trachomatis* infection rate of 312.7 per 100,000 population) revealed that repeat testing versus performance of the APTIMA CT assay on AC2-positive *C. trachomatis* screens demonstrated 95% concordance of the final result [114]. Schachter et al. [106] utilized a moderate-prevalence California population (336.7 cases per 100,000 population) to demonstrate an 84–98% rate of initial AC2 screen confirmation by repeat testing and a potentially elevated rate (89–99%) of initially positive screens confirmed by secondary NAAT. In a high-prevalence population (684.0 cases per 100,000 population), significantly more low-positive *C. trachomatis* screens were confirmed by the APTIMA CT assay than by duplicate repeat testing [109]. However, these authors noted that utilization of alternative target TMA for confirmation raised overall AC2 *C. trachomatis* positive predictive value only 1.8% over that derived from repeat testing. Similar findings characterized *N. gonorrhoeae* screen verification algorithms. Zanto et al. [114] demonstrated 90% concordance in final *N. gonorrhoeae* AC2 result derived by repeat testing versus alternative target TMA. Moncada et al. [87] summarized their side-by-side comparison of the two advocated methods by noting that 89–96% of specimens positive by initial NAAT were confirmed by repeat testing and that 85–98% of initial screening results were confirmed via secondary NAAT. In a high-prevalence gonorrhea population (265.9 cases per 100,000 population), confirmatory testing via the APTIMA GC assay demonstrated only a 5% increase in the rate of AC2 low-positive result retention [109].

## 6. Test of Cure

Due to very high microbial cure rates exhibited by azithromycin and doxycycline in a recent meta-analysis [115], the CDC does not advocate *C. trachomatis* test-of-cure analysis in males or in nongravid females, unless therapeutic compliance is questioned, symptoms persist, or reinfection is suspected [116]. Workowski et al. [117] utilized an in-house PCR to demonstrate a reduction in rate of endocervical detection of *C. trachomatis* from 50% immediately following completion of doxycycline therapy to 15% seven days later. Nucleic acid was not detected at the two-week interval. Gaydos et al. [118] reported greater *C. trachomatis*-specific nucleic acid recovery rates for commercial ligase chain reaction (37–73%, interval dependent) over those of AMP (21–40% for similar intervals) within the first six days following the completion of therapy.

In contrast, an in-house PCR detected *C. trachomatis* nucleic acid from 25% of endocervical swabs collected three weeks after the completion of therapy [119]. In the same study, nucleic acid sequence-based amplification, an RNA amplification technology, yielded a *C. trachomatis* detection rate of only 6.7% and 8.0% from urine and endocervical specimens, respectively, one week after the completion of therapy. Comparator percentages were 26.7% and 84.0% for the PCR. Bianchi et al. [120] subjected post treatment urine specimens to AMP and the Gen-Probe first generation TMA assay. Kinetics of both systems was essentially equivalent in females, demonstrating full clearance within six days. Similar results were generated in a smaller sampling of males.

According to recently published CDC recommendations [116], test-of-cure protocols are unnecessary for patients who have completed antimicrobial therapy for *N. gonorrhoeae* infection because multiple lines of therapy have proven efficacious [121]. Exceptions to this paradigm are in the minority and are potentially linked to increasing resistance of *N. gonorrhoeae* to fluoroquinolone agents [122, 123]. However, high prevalence of *N. gonorrhoeae* infection exists in patients who have had gonorrhea in the preceding months [124]. These data imply that the detection of *N. gonorrhoeae* post-treatment may actually be reflective of reinfection rather than treatment failure. If symptoms persist in patients following the completion of therapy, clinicians may consider re-testing patients, via culture modalities, for the ultimate purposes of antimicrobial susceptibility testing. This approach is hypothetically far more efficacious in the management of *N. gonorrhoeae* infection than in *C. trachomatis* infection because of stark differences in culture sensitivity [28].

A paucity of studies has characterized an auxiliary role for NAAT in gonorrhea test-of-cure. For example, Hanks et al. [125] demonstrated that nucleic acid hybridization was unable to generate *N. gonorrhoeae* signal from genital and urine specimens of patients between six and eleven days post-completion of antimicrobial therapy for *N. gonorrhoeae* infection. Bachmann et al. [126], utilizing a commercial ligase chain reaction, reported the median time to a negative *N. gonorrhoeae* urine assay being one day for males and two days for females upon completion of therapy. Among females, the mean clearance time proved greater for genital specimens (2.8 days) than for urine specimens (1.7 days; *P* = 0.008). An intermittent shedding phenomenon was observed in 15% of males and 25% of females during the three-week follow-up period. Women who shed *N. gonorrhoeae* nucleic acid intermittently were twice as likely to have a genital specimen yield detectable *N. gonorrhoeae* compared to a urine specimen. In one female patient, such detection occurred 19 days after the completion of therapy.

## 7. Utility in Medicolegal Settings

The shorter length of the vagina in prepubescent girls, combined with its columnar epithelial cell lining and alkaline environment, can predispose this population to infection with sexually transmitted agents including *C. trachomatis* [127]. The extrapolation of *C. trachomatis* detection in children beyond the neonatal period to sexual abuse [116] has some limitations. It has been estimated that 20% of infants born to women with active *C. trachomatis* infection can acquire the infection in rectal and vaginal sites [128]. Persistence of the organism acquired in perinatal fashion may last 2-3 years [129]. Moreover, retrospective chart reviews [130–133], subcomponents of which utilized *C. trachomatis* NAAT, have outlined very low incidence of *C. trachomatis* detection (0.5–3.1%) in the context of child sexual abuse. Schachter et al. [79], within a significant at-risk population for STI acquisition, reported lower *C. trachomatis* detection rates from oropharyngeal and rectal sites (0.8% and 6.1%, resp.) than those for *N. gonorrhoeae*. Taken together, it must be noted that the positive predictive value of even very highly specific NAAT screens can be compromised by low disease prevalence in a given setting [134].

Largely as a result, it has been a long-standing axiom that *C. trachomatis* cultivation has more validity in medicolegal proceedings than results derived from NAAT [135]. Despite this, in a limited sexual abuse victim dataset, Matthews-Greer et al. [136] described 11 instances of *C. trachomatis* culture data being corroborated by a positive PCR result. Two rectal specimens were culture positive/PCR negative, while one rectal and three female genital swabs yielded culture-negative/PCR-positive results. Girardet et al. [137] reported that 18 of 215 (8.4%) possible pediatric sexual abuse victims generated a positive NAAT for *C. trachomatis* from a noninvasive urine specimen. Cultures for *C. trachomatis* were positive in only 44% of instances. Kellogg et al. [138] reported 11–18% agreement between *C. trachomatis* culture results and those derived from ligase chain reaction or PCR performance on urine and vaginal specimens collected from girls who reported abusive sexual contact. Among 485 girls being evaluated for sexual abuse, sensitivity of urine *C. trachomatis* NAAT was 100% when compared to vaginal culture [139]. Eight additional patients yielded positive urine *C. trachomatis* NAAT in the face of negative culture (*P* = 0.018).

Interestingly, in 2001 Hammerschlag [134] stated that the advancement of NAAT evidence in courts of law was hindered by the paucity of appropriate commercially available confirmatory assays. With the advent of the APTIMA CT assay and defined performance characteristics [32, 112], perhaps this scenario warrants additional consideration. CDC recommendations have varied on this topic within the past five years. 2006 recommendations for the management of STI [140] stated that NAAT might be a viable alternative in the detection of *C. trachomatis* if culture systems for the organism are unavailable and if a method of confirmation is available. Noted means of confirmation included secondary FDA-cleared NAAT targeting a different sequence than the primary screening method [140]. This nonculture option for the detection was not advocated for laboratory diagnosis of *N. gonorrhoeae*. Black et al. [139] remarked that urine NAAT methodologies (with subsequent confirmatory testing) are adequate as a new forensic standard in children suspected of being sexual abuse victims. The recently published guidelines [116] place primary focus on standardized anal (both genders), urethral discharge (males), and vaginal culture techniques for both *C. trachomatis* and *N. gonorrhoeae* (Table 3). The paucity of specimen source options available for *C. trachomatis* culture is related to low source-specific organism prevalence rates and the paradigm of chlamydial persistence following perinatal acquisition. NAAT for *C. trachomatis* and *N. gonorrhoeae* from vaginal and urine specimens is recommended in girls as an alternative to culture. Furthermore, in the context of sexual assault in adults and adolescents, the latest recommendations call for the performance of FDA-cleared NAAT for either *C. trachomatis* or *N. gonorrhoeae* upon initial examination. The topic of STI detection in the context of medicolegal testing has been reviewed extensively by Hammerschlag and Guillén [141].

## 8. Conclusions

Independent of specimen transport conditions, sensitivity of *C. trachomatis* culture is greatly inferior to those of amplified molecular methods that have since largely replaced signal amplification and nucleic acid hybridization assays. Because molecular-based testing for *N. gonorrhoeae* is simultaneously provided within commercial molecular assays for *C. trachomatis*, many laboratories subsequently forego sole reliance upon culture methods for *N. gonorrhoeae* detection from urine and genital sources. Sensitivity differences between commercial PCR, SDA, and TMA have been delineated in the literature, both in clinical and *in vitro* settings. The aforementioned specimen types are applicable to AC2, with the addition of vaginal swab and gynecological specimens (Table 4). A limited role for *C. trachomatis* culture may be seen in medicolegal settings or for cultivation from specimen sources that are not indicated for FDA-approved NAAT. However, studies have emerged advocating a highly sensitive NAAT modality, such as commercial TMA, to augment culture methodology for accurate detection of *C. trachomatis* from extragenital sites.

Validity of results generated by highly sensitive modalities has been addressed with a variety of confirmatory testing algorithms. Limitations to follow-up testing include clinicians not routinely providing two specimens for evaluation and the prohibitive expense for some laboratories to either modify an existing molecular assay to target a different nucleic acid sequence or validate secondary NAAT. Even when NAAT is utilized as a means of confirmation, differences in performance characteristics of these assays, deficiencies in result reproducibility for a given specimen using the same testing modality, and potential differences in sensitivity related to heterologous specimen collection media/transport devices have been reported [106]. Even when repeat testing is factored into this paradigm, additional generated data may be difficult to interpret, especially when considering the extremely high rates of sensitivity and specificity already inherent to these screening assays. It must be kept in mind that CDC recommendations related to *C. trachomatis* screening diagnostics have not been updated since 2002 [29]. On the basis of the discussion provided in Section 5 of this review, any subsequent revision may result in significant changes related to this paradigm.

While currently not widely accepted as medicolegal evidence due to concerns over specificity, admission of NAAT results may eventually become standard practice in courts of law. Prominent acceptance of alternative target confirmatory testing may have to play a significant role for this to occur, particularly with a highly sensitive method such as commercial TMA. Viable test-of-cure options are not extensive in the setting of chlamydia due to meager sensitivity of *C. trachomatis* culture. At the same time, amplified molecular test-of-cure protocols are deemed unnecessary in a majority of settings due to efficacious therapeutic regimens. Auxiliary studies utilizing NAAT demonstrate microbiological cure approximately 7–14 days after therapy, yet investigations in this vein using latest-generation commercial TMA would be compelling.

Chlamydia prevalence has experienced a significant upswing in the United States over the past 15 years. Clinical presentation of male urethritis exhibits overlap with that of nongonococcal urethritis. In females, symptoms of chlamydia can resemble those of gonorrhea or trichomoniasis. These data predicate the importance of laboratory detection of *C. trachomatis*. Poor *C. trachomatis* culture sensitivity signifies the importance of nonculture diagnostic modalities. With respect to the utilization of the rapid, nonamplification methods for *C. trachomatis* detection, one must be cautious to the actual “point-of-care” benefit therein, as many studies have proven these methods to have much lower analytical sensitivity than NAAT. Consequently, as nucleic acid-based diagnostic assays continue to improve, a greater presence for such testing needs to be established in both small- and large-scale clinical laboratory settings.

## Figures and Tables

**Table 1:** Factors that attempt to distinguish male nongonococcal urethritis from those cases caused by *Neisseria gonorrhoeae*.

Factor	Gonococcal urethritis	Nongonococcal urethritis	Reference(s)
Typical incubation period	2–5 days	7–14 days	[14, 15]
Range of symptom onset	1–10 days	2–35 days	[14, 15]
Frank purulent discharge (% of cases)	75	11–33	[16, 17]
Mucopurulent discharge (% of cases)	25	50	[16, 17]
Clear or moderately viscid discharge (% of cases)	4	10–50	[16, 17]
Dysuria (% of cases)	73–88	53–75	[16, 18]
Combination of dysuria and discharge (% of cases)	71	38	[16]

**Table 2:** Performance characteristics of three commercially-available *Chlamydia trachomatis *molecular screening platforms per manufacturer-provided data. Ranges reflect differences in performance characteristics between specimens collected from symptomatic and asymptomatic individuals (when specified).

Commercial assay	Gender	Specimen	Performance indices	
			Sensitivity range (%)	Specificity range (%)
AMPLICOR	Female	Endocervical	87.4–94.0	98.6
		Urine	84.3–89.5	98.0–98.8
	Male	Urethral	96.3–98.7	95.2–97.7
		Urine	87.6–92.0	91.9–95.7

BD ProbeTec ET	Female	Endocervical	88.7–96.8	97.9–98.5
		Urine	77.0–83.9	98.2–98.3
	Male	Urethral	89.5–95.5	92.9–97.0
		Urine	89.5–95.4	89.4–95.8

APTIMA Combo 2^†^	Female	Endocervical	92.4–98.4	96.7–98.8
		Urine	93.8–96.8	98.8–99.0
	Male	Urethral	94.6–96.4	96.9–98.4
		Urine	96.3–98.5	98.4–98.8

^†^APTIMA Combo 2 clinician-collected vaginal swab sensitivity range 96.5–96.7%, specificity range 96.4–97.2%; APTIMA Combo 2 patient-collected vaginal swab sensitivity 98.4%, specificity 96.8%.

**Table 3:** Recommended diagnostic modalities and specimen sources for evaluation of child sexual abuse victims. Data are adapted from [116].

Diagnostic modality	Recommended specimen sources for STI etiology			
	*Chlamydia trachomatis*		*Neisseria gonorrhoeae*	
	Female	Male	Female	Male
			Oropharynx	Oropharynx
	Anus	Anus	Anus	Anus
Culture				Urethra
		Urethral discharge		Urethral discharge
	Vagina		Vagina	

NAAT^†^	Urine		Urine	
	Vagina		Vagina	

^†^Nucleic acid amplification testing.

**Table 4:** United states food and drug administration-approved specimen sources for commercially available *Chlamydia trachomatis* nucleic acid amplification testing. Data are derived from [50–52].

Modality	Symptomatic female	Symptomatic male	Asymptomatic female	Asymptomatic male
AMPLICOR	Endocervix	Urethra	Endocervix	Urethra
	Urine	Urine	Urine	Urine

BD ProbeTec ET	Endocervix	Urethra	Endocervix	Urethra
	Urine	Urine	Urine	Urine

APTIMA Combo 2	Endocervix	Urethra	Endocervix	Urethra
	Urine	Urine	Urine	Urine
	Vagina^a^		Vagina^a^	
	Gynecology Exam^b^		Gynecology Exam^b^	
			Vagina^c^	

^a^Clinician collected.

^b^Collected in PreservCyt Solution; processed with ThinPrep 2000 system (Cytyc Corporation, Marlborough, Mass, USA).

^c^Patient collected.
